# Editorial: Pain in multiple sclerosis and experimental autoimmune encephalomyelitis

**DOI:** 10.3389/fneur.2022.1093254

**Published:** 2022-11-22

**Authors:** Amy Lovett-Racke, David Henrique Rodrigues, Gila Moalem-Taylor

**Affiliations:** ^1^Department of Microbial Infection and Immunity, College of Medicine, The Ohio State University, Columbus, OH, United States; ^2^Department of Basic Life Sciences, Institute of Life Sciences, Federal University of Juiz de Fora, Governador Valadares, Brazil; ^3^Neuropathic Pain Research Group, Translational Neuroscience Facility, School of Biomedical Sciences (SBMS), The University of New South Wales, UNSW Sydney, Sydney, NSW, Australia

**Keywords:** multiple sclerosis, experimental autoimmune encephalomyelitis, neuropathic pain, neuroinflammation, demyelinating disease

Multiple sclerosis (MS) is a chronic inflammatory demyelinating disease of the central nervous system, associated with a substantial social and financial burden in many countries. Sensory symptoms are among the first clinical manifestations, and patients often present with acute or chronic pain of varying degrees—from mild to debilitating. Not surprisingly, a growing body of research seeks to unveil the molecular and cellular mechanisms that lead to pain in MS with the goal of developing effective pain management treatments.

In preclinical research, one of the most widely used animal models that allow the investigation of pain in MS is experimental autoimmune encephalomyelitis (EAE). Animals with EAE develop a neuroinflammatory condition that leads to demyelination, glial cell activation, and neuro-axonal injury, which mimic MS and is associated with acute and chronic neurological deficits—hallmarks of MS. Several studies utilizing different experimental pain assessments demonstrated that pain hypersensitivity constitutes one of the early signs of EAE animals. Despite recent advances in the field, the mechanisms underlying such pain in EAE and MS remain incompletely understood. Thus, in this Research Topic, we aimed to discuss the various pain syndromes associated with MS and their pathophysiology, the use of animal models and the implicated mechanisms, as well as knowledge gaps and future research directions for the study of pain in MS.

Typically, people who work in preclinical research have limited contact with patients, and rely on books and papers to understand how pain is elicited in MS. The paper from Racke et al. brings us an excellent perspective on pain manifestations in real patients. The clinical vignettes are a handy resource for getting familiar with the various pain syndromes in MS (e.g., neuropathic pain, Lhermitte's phenomenon, trigeminal neuralgia, optic neuritis pain, and muscular pain), especially for those away from the bedside. This article demonstrates that pain experiences can appear in distinct and occasionally unusual ways. Additionally, the examples of pain in MS patients illustrate that pain is complex, affects the emotional wellbeing of sufferers, and is highly associated with reduced quality of life, necessitating more research and therapies.

Both nociceptive and neuropathic pain are prevalent in MS patients. As previously mentioned, the mechanisms underlying the pain observed in MS and EAE are still poorly understood. Mirabelli and Elkabes review neuropathic pain, the role of glial cells and glial-neuronal interactions, and the ion channels, pumps, and exchangers involved in pain signaling in EAE. The paper also highlights the potential hyperalgesic role of some immune mediators, such as chemokines, which is crucial in understanding unique pain aspects in MS.

Of the many immune mediators that are increased in the central nervous system in both patients with MS and animals with EAE, the tumor necrosis factor-alpha (TNF-α) seems to play a pivotal role. Maguire et al. review the role of TNF-α in neuroinflammation in both EAE and MS, elaborating on how TNF-α contributes to the sensitization of peripheral nociceptors and spinal dorsal horn neurons and pain. This study highlights how TNF-α function is distinct and can lead to different outcomes (on the one hand, cell death *via* apoptosis or necrosis, or on the other hand, survival and inflammation) based on the isoform and its signaling *via* TNFR1 or TNFR2 pathway. While there is significant evidence that TNF-α contributes to pain in EAE and MS, there is a need to elucidate these pathways further and develop more specific inhibitors to the TNF-α isoform and TNF receptor types.

One of the drawbacks of EAE models is the use of complete Freund adjuvant for EAE induction since its use may also result in disadvantages that include inducing neuroinflammatory changes and severe motor symptoms. This is challenging for researchers because many laboratory tools that measure “pain” in animals depend on their motor skills. Thus, Démosthènes et al. comprehensively characterize an EAE model that uses QuilA as an adjuvant, which was developed initially in 2007, and was then assessed for pain behaviors in 2014. Démosthènes et al. extend the findings and demonstrate that QuilA-EAE mice develop long-lasting mechanical allodynia, heat hyperalgesia, and cold allodynia, with no motor impairment, but do not develop orofacial hypersensitivity and cognition and anxiety changes. Treatment with the drug pregabalin had an analgesic effect, validating the model for the study of pain in EAE.

In summary, this Research Topic discusses key concepts in the field of pain in EAE and MS. We expect this issue to help researchers in their future studies to investigate pain in EAE animals, in MS patients, and to those who are curious and want to deepen their knowledge on this topic. The work presented in this Research Topic also emphasizes the need for further preclinical and clinical studies on MS pain.

## Author contributions

All authors listed have made a substantial, direct, and intellectual contribution to the work and approved it for publication.

## Conflict of interest

The authors declare that the research was conducted in the absence of any commercial or financial relationships that could be construed as a potential conflict of interest.

## Publisher's note

All claims expressed in this article are solely those of the authors and do not necessarily represent those of their affiliated organizations, or those of the publisher, the editors and the reviewers. Any product that may be evaluated in this article, or claim that may be made by its manufacturer, is not guaranteed or endorsed by the publisher.

